# BMS309403 Stimulates Glucose Uptake in Myotubes through Activation of AMP-Activated Protein Kinase

**DOI:** 10.1371/journal.pone.0044570

**Published:** 2012-08-31

**Authors:** Wanhua Lin, Xiaoli Huang, Lina Zhang, Dongmei Chen, Dongye Wang, Qilong Peng, Lei Xu, Jingya Li, Xiujie Liu, Kuai Li, Ke Ding, Shouguang Jin, Jia Li, Donghai Wu

**Affiliations:** 1 The Key Laboratory of Regenerative Biology, Guangzhou Institutes of Biomedicine and Health, Chinese Academy of Sciences, Guangzhou, China; 2 National Center for Drug Screening, State Key Laboratory of Drug Research, Shanghai Institute of Materia Medica, Chinese Academy of Sciences, Shanghai, China; 3 Institute of Chemical Biology, Guangzhou Institutes of Biomedicine and Health, Chinese Academy of Sciences, Guangzhou, China; 4 Department of Molecular Genetics and Microbiology, University of Florida, Gainesville, Florida, United States of America; University of Hong Kong, China

## Abstract

BMS309403 is a biphenyl azole inhibitor against fatty acid binding protein 4 (FABP4) and regarded as a lead compound for effective treatment of obesity related cardio-metabolic diseases. Here we discovered an off-target activity of BMS309403 in that it stimulates glucose uptake in C2C12 myotubes in a temporal and dose dependent manner via activation of AMP-activated protein kinase (AMPK) signaling pathway but independent of FABPs. Further analysis indicated that BMS309403 activates AMPK through increasing the ratio of intracellular AMP:ATP while decreasing mitochondrial membrane potential. These findings provide mechanistic insights on the action of BMS309403.

## Introduction

BMS309403 is a biphenyl azole inhibitor specifically designed to target FABP4 with a Ki value less than 2 nM [1]. Mice orally administered with BMS309403 are effectively protected against severe atherosclerosis and type 2 diabetes [2]. Mechanistically, BMS309403 inhibits lipid accumulation, cholesterol efflux and inflammatory responses in macrophages, and suppresses fatty acid uptake in adipocytes, in a FABP4-dependent manner [2]. Although FABP4 is predominantly present in macrophages and adipocytes, its expression has also been detected in other tissues such as endothelial cells [3] and human muscles [4]. Notably, in cultured human microvascular endothelial cells, lipid-induced impairment on eNOS phosphorylation and NO production could be readily reversed by BMS309403 [3]. However, whether BMS309403 has direct effects on muscle cells has not been reported. Muscle is the primary peripheral tissue responsible for glucose uptake and defect in glucose uptake in skeletal muscle is one of the major pathogenic features in both type 1 and type 2 diabetes [5]. In muscle, glucose uptake is stimulated by either insulin or muscle contraction. While PI3K/Akt signaling pathway is mainly responsible for evoked Glut4 translocation and glucose utilization [6], AMP-activated protein kinase (AMPK) plays a predominant role in stimulating glucose uptake during exercise via activation of p38 MAPK [7], [8]. AMPK is an evolutionarily conserved heterotrimeric serine/threonine kinase which acts as a “Fuel Gauge” that maintains the cellular as well as body energy balance by sensing the intracellular AMP/ATP ratio [9]. In response to a fall in intracellular ATP level, AMPK activates energy-producing processes such as glucose uptake, fatty acid oxidation and glycolysis, while inhibiting energy-consuming processes such as lipogenesis, protein synthesis and gluconeogenesis [10].

In the present study, we found that BMS309403 enhances glucose uptake in myotubes through activation of the AMPK signaling pathway, which was accompanied by altered mitochondrial membrane potential and AMP/ATP ratio. These actions are at least partially independent of FABPs. Our findings provide new mechanistic insights into the actions of BMS309403 and this class of pharmacological reagents in combating obesity and related metabolic diseases.

## Results

### BMS30943 Stimulates Glucose Uptake, AMPK and p38 Phosphorylation in Differentiated C2C12 Myotubes

**Figure 1:**
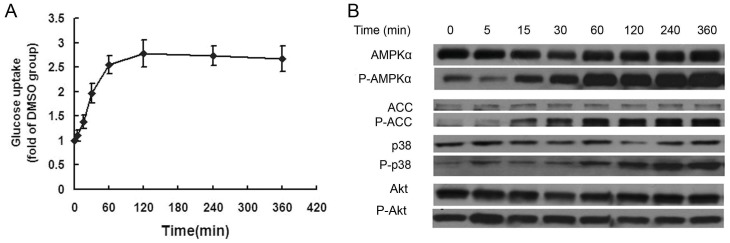
BMS309403 stimulates glucose uptake and phosphorylation of AMPK and p38 in a time dependent manner. C2C12 myotubes were starved for 2 hours in serum free high glucose DMEM containing 0.2% BSA followed by incubation with 20 µM BMS30943 for various time periods as indicated. (A) Glucose uptake by C2C12 myotubes. The results were expressed as fold over DMSO group; (B) Western blotting analysis of AMPK signaling pathway and Akt in C2C12 cells.

BMS309403 was previously reported to increase glucose uptake in adipocytes [2]. In order to examine whether this compound has any biological effects on muscle cells, differentiated C2C12 myotubes were incubated with 20 µM BMS309403. We found that BMS309403 increased glucose uptake in C2C12 myotubes in a time-dependent manner (Fig. 1A). The incremental effect reached maximum 2 hours after incubation with BMS309403 where nearly 3 fold increase was observed compared with the control. To further characterize this process and the underlying mechanisms, activation of AMPK and Akt upon BMS309403 treatment was examined. The results demonstrated that BMS309403 caused a remarkable activation of the AMPK, as determined by phosphorylation of AMPKα on Thr-172 (Fig. 1B). In parallel, phosphorylation of p38, the key effector kinase responsible for the AMPK related glucose uptake, was also evident in C2C12 myotubes upon incubation with BMS309403 (Fig. 1B). The enhancement of AMPK activity was also demonstrated by the phosphorylation of acetyl-CoA carboxylase (ACC), which is one of the best-known downstream targets of AMPK. In contrast, Akt was not activated by BMS309403 treatment (Fig. 1B).

**Figure 2:**
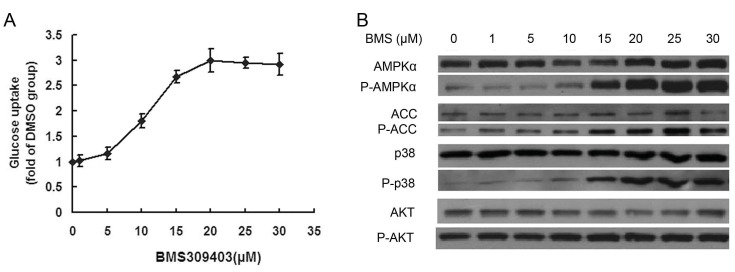
BMS309403 stimulates glucose uptake in C2C12 myotubes and AMPK signaling pathway in a dose dependent manner. C2C12 myotubes were starved for 2 hours in serum free high glucose DMEM containing 0.2%BSA followed by incubation for 2 h with the indicated concentrations of BMS309403. (A) 2-deoxy-D[1-^3^H]-glucose was added for 15 min at 37°C, followed by glucose uptake measurement. (B) Western blotting analysis of AMPK and Akt signaling molecules.

BMS309403 increased glucose uptake in a dose dependent manner up to 20 µM with a maximal stimulatory effect at 20 µM (Fig. 2A). Likewise, BMS309403 increased phosphorylation of AMPKα on Thr-172 in a similar fashion where AMPKα phosphorylation reached a maximum level at 20 µM (Fig. 2B). In parallel with the increased phosphorylation of AMPKα, the phosphorylation of p38 and ACC was also increased in a dose dependent manner (Fig. 2B).

**Figure 3:**
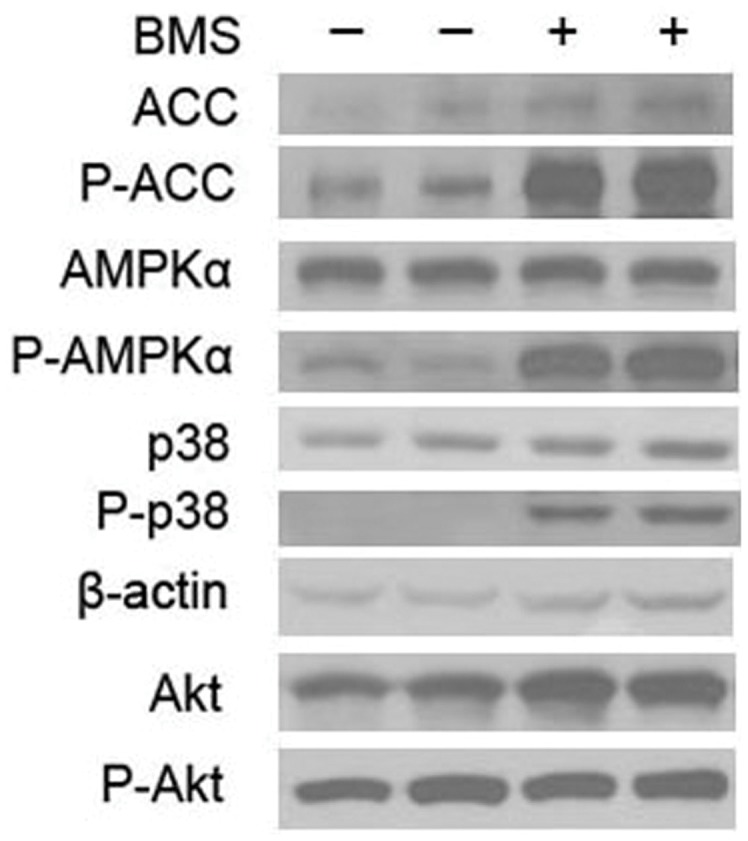
Western blotting analysis of AMPK signaling pathway following treatment with BMS309403 in L6 myotubes. L6 myotubes were starved for 2 hours in serum free high glucose DMEM containing 0.2%BSA followed by incubation for 2 h with 30 µM of BMS309403.

To determine whether BMS309403 has the same effect on muscle cells other than the C2C12, rat L6 cells were tested. L6 cells were cultured and differentiated into myotubes, as described in *Materials and Methods*, before treated with BMS309403. As shown in Figure 3, western blotting analysis demonstrated that BMS309403 activated AMPK signaling pathway by increasing the phosphorylation of AMPK, p38 and ACC while not affecting Akt (Fig. 3).

### BMS309403 Stimulates Glucose Uptake in C2C12 Myotubes Via AMPK Activation

**Figure 4:**
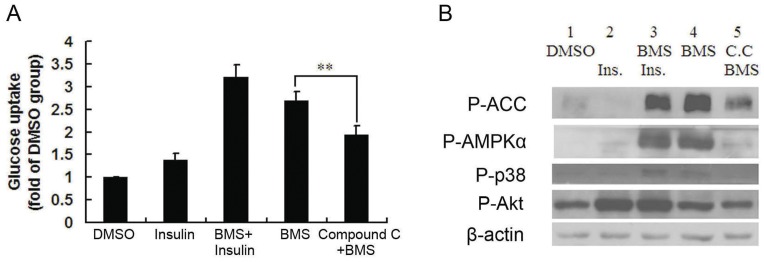
AMPK activation is indispensable for BMS309403 induced glucose uptake. C2C12 myotubes were pretreated with compound C (C.C) for 30 min followed by the addition of BMS309403 and/or insulin. (A) Glucose uptake in C2C12 myotubes following indicated treatments. (B) Western blotting analysis of related signaling molecules in C2C12 myotubes.

The above results indicate that BMS309403 induced enhancement of glucose uptake was accompanied by activation of AMPK. To further evaluate whether AMPK is directly responsible for the BMS309403-induced glucose uptake in myotubes, C2C12 myotubes were pre-incubated with or without an AMPK inhibitor, compound C, before the addition of BMS309403. Insulin was also added alone or in combination with BMS309403 as a control. As shown in Figure 4A, either BMS309403 or insulin alone increased glucose uptake. Western blotting analysis confirmed that BMS309403 evoked phosphorylations of AMPKα, p38 and ACC were remarkably attenuated upon pretreatment with the compound C (Fig. 4B). Notably, an additive effect of BMS309403 with insulin on glucose uptake was observed (Fig. 4), suggesting that these two reagents work through distinct signaling pathways. More importantly, compound C significantly suppressed BMS309403 stimulated glucose uptake (Fig. 4). Taken together, these data indicated that BMS309403 stimulated glucose uptake via an AMPK-dependent pathway but independent of insulin signaling in C2C12 myotubes.

### BMS309403 Activates AMPK Independent of FABP3

**Figure 5:**
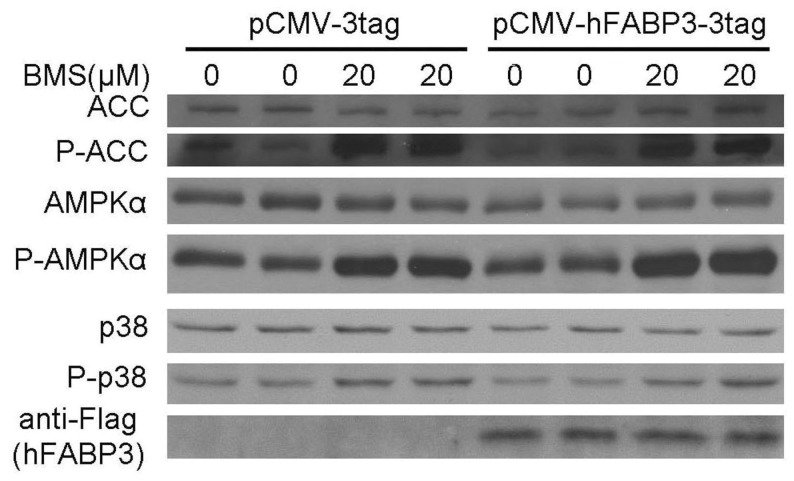
AMPK activation by BMS309403 is independent of hFABP3. Western blotting analysis of AMPK phosphorylation in C2C12 myobubes overexpressing FABP3 with or without BMS309403 treatment.

BMS309403 was designed as a competitive inhibitor not only for FABP4 but also for FABP3 and FABP5, exhibiting Ki values<2 nM for FABP4, compared with these of 250 nM for FABP3 and 350 nM for FABP5 [1]. FABP3 was the predominant form of FABP in myotubes. We next examined whether the enhancement of the AMPK activity by BMS309403 in muscle cells requires FABPs. To this end, the possible effect of overexpression of human FABP3 on BMS309403 associated AMPK activity was examined. Stable C2C12 cell lines overexpressing human FABP3 were established by either pCMV-3tag with the empty vector as a negative control. These cells can be differentiated to myotubes normally and the differentiated C2C12 myotubes were treated with BMS309403. Western blotting analysis demonstrated that overexpression of hFABP3 did not alter the phosphorylation of AMPKα, either in absence or presence of BMS309403 (Fig. 5).

**Figure 6:**
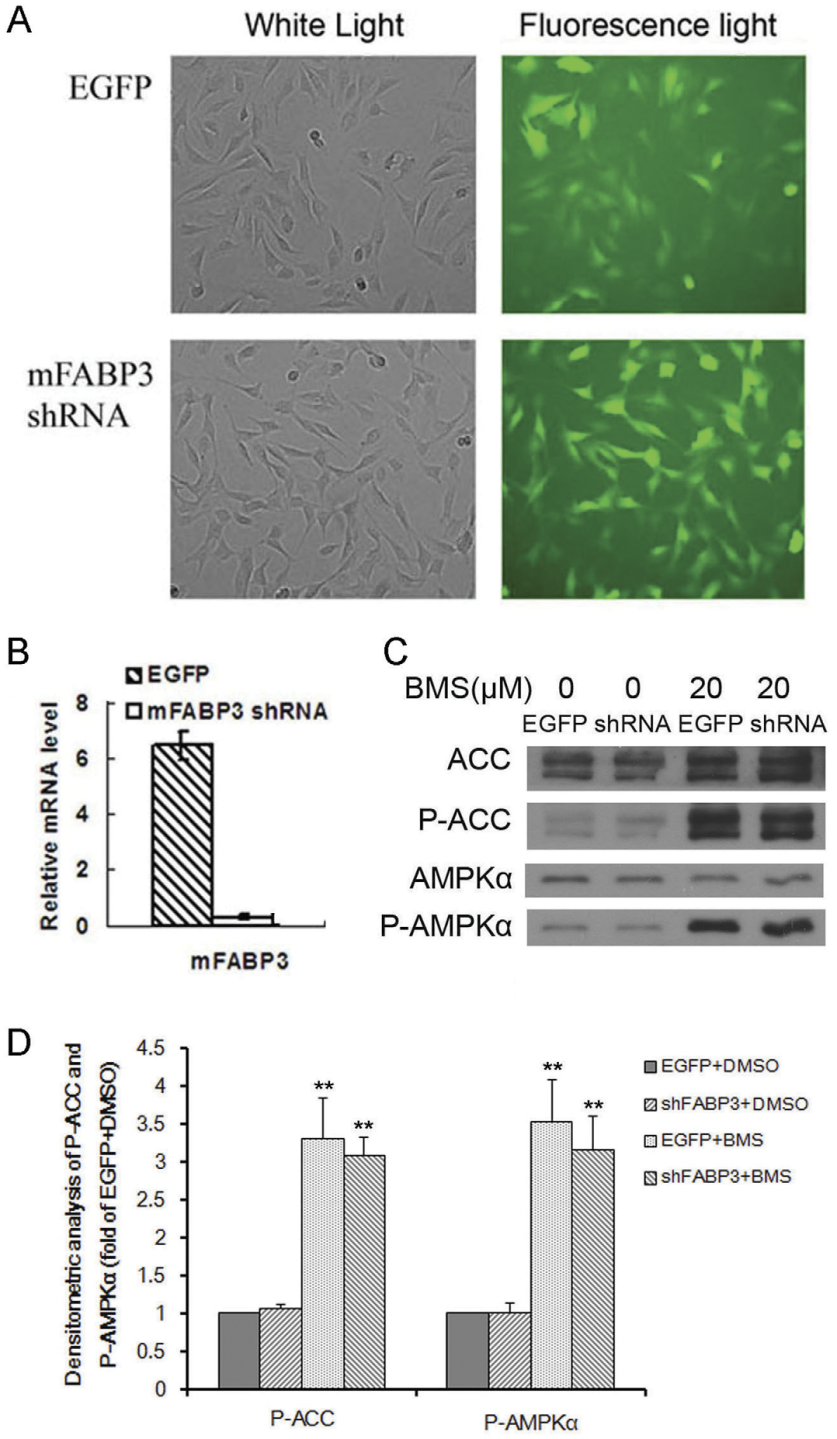
BMS309403 triggered AMPK activation remains intact upon knockdown of endogenous FABP3 in C2C12 myotubes. (A) Microscopy photography (×100) of C2C12 cell lines stably expressing lentiviral HIVU6-EGFP or HIVU6-shRNA against mFABP3 (shFABP3). (B) Quantitative real-time PCR analysis of FABP3 gene expression in differentiated C2C12 stable myotubes. (C) Western blotting analysis of AMPK signaling pathway following BMS309403 treatment in the control and FABP3 knocked-down C2C12 myotubes. (D) Densitometric analysis of phospho-ACC and phospho-AMPKα in C2C12 stable myotubes of EGFP or shFABP3 treated with DMSO or 20 µM BMS309403. C2C12 stable myotubes of EGFP treated with DMSO was used as control. There was significant difference between DMSO and BMS group, but no significant difference between EGFP + BMS and shFABP3+ BMS group.

Furthermore stable C2C12 transfectants expressing shRNAs against mouse FABP3 were established following lentiviral transfection and selection with puromycin (Fig. 6A). Resulting stable cell lines could be differentiated to myotubes as the parent C2C12 cells. Quantitative real-time PCR analysis showed that lentivirus-mediated RNA interference against FABP3 resulted in up to ∼95% silencing of the FABP3 compared with the control cell line expressing GFP only (Fig. 6B). The effects of BMS309403 on the above cell lines were further evaluated and compared. The results showed that BMS309403 mediated AMPK activation was not mitigated upon knockdown of the endogenous FABP3 in C2C12 myotubes (Fig. 6C and Fig. 6D).

### AMPK is not Directly Activated by BMS309403 *in vitro*

**Figure 7:**
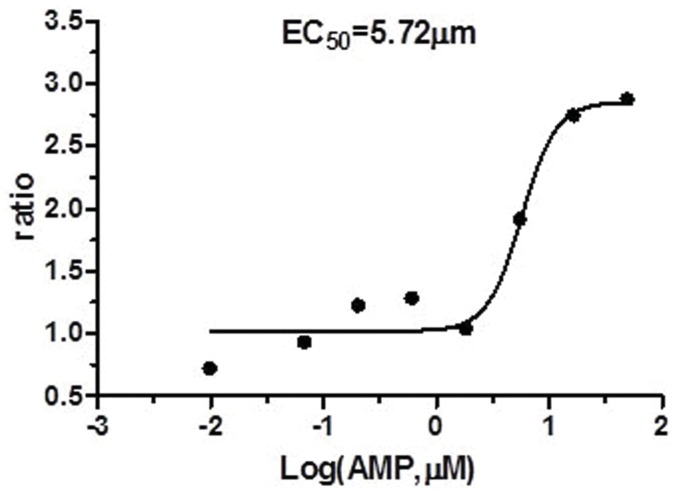
BMS309403 is not a direct activator of AMPK. Results of *in vitro* AMPK assay for AMPKα2β1γ1 incubated with BMS309403 or AMP.

To assess whether BMS309403 is a direct activator of AMPK, an AMPK activity assay was performed. The typical form of heterotrimeric AMPK, *i.e.* AMPKα2β1γ1 [11], was used. 40 µM of BMS309403 was co-incubated with SAMS peptide as substrate, and AMPK activity was evaluated by the incorporation of [γ-^33^P] into the SAMS peptide [12]. AMP, as an allosterical activator of AMPK, was used as a positive control. AMP increased AMPK activity by about 2-fold with an EC_50_ of about 5 µM (Table 1 and Fig. 7). However, BMS309403 failed to cause any significant phospho-incorporation into SAMS peptide (Table 1), demonstrating that AMPK was not directly activated by BMS309403.

**Table 1:** AMPKα_2_β_1_γ_1_ Activity by AMP and BMS309403.

Compound name	Concentration	Result type	Result unit	Result	Error
AMP		EC50	µM	5.72	
BMS309403	20µg/mL	%Activity	percentage	82.34	2.02

### BMS309403 Depolarizes Mitochondrial Membrane Potential and Increases Cytosolic AMP/ATP Ratio

**Figure 8:**
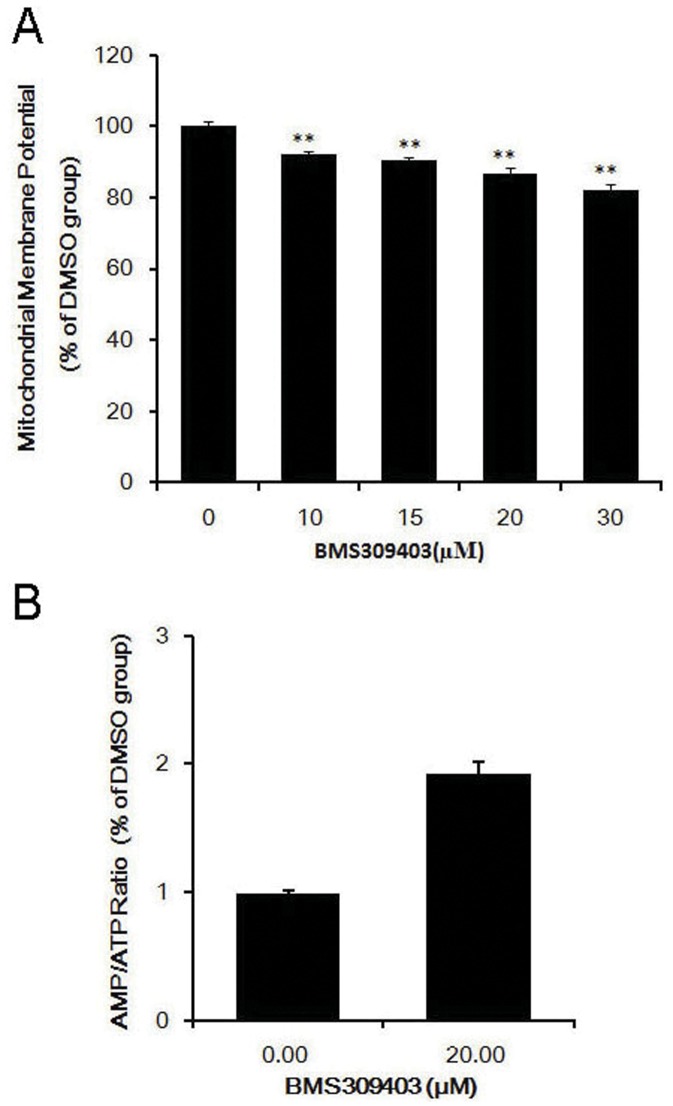
BMS309403 reduces mitochondrial membrane potential in a dose-dependent manner and increases cytosolic AMP/ATP ratio in C2C12 myotubes. (A) Mitochondrial membrane potentials of C2C12 myotubes were measured on day 5 of differentiation after pretreated with BMS309403 at 37°C for 2hours. (B) Adenine nucleotides from perchloric acid extracts of C2C12 myotubes were measured by HPLC. Values were means ± SE for three independent experiments. **p<0.01 vs DMSO.

Mitochondrial membrane potential is built up by oxidative phosphorylation which is coupled to ATP production. Uncoupling of the oxidative phosphorylation results in a depolarized mitochondrial membrane potential and subsequently causes a decrease in ATP level. Therefore, we next sought to address whether BMS309403 acts as a chemical uncoupler to modulate intracellular AMPK activity. The lipophilic cationic probe JC-1 (5,5′,6,6′-tetrachloro-1,1′,3,3′-tetraethylbenzimi-dazolyl -carbocyanine iodide ) is a well-developed indicator of mitochondrial membrane potential. C2C12 myotubes were treated with DMSO or different concentrations of BMS309403. The mitochondrial membrane potential was measured using the dye JC-1. As shown in Figure 8A, BMS309403 decreased mitochondrial membrane potential in a dose-dependent manner. A significant decrease (∼13%) of Δψm was observed when C2C12 myotubes were treated with 20 µM BMS309403 for 2 hours.

Whether BMS309403 increased ratio of AMP to ATP was investigated as well. To do this, adenine nucleotide in C2C12 myotubes was measured by HPLC following treatment with DMSO or 20 µM BMS309403. Consistent with the uncoupling effect observed for BMS309403, the AMP: ATP ratio was increased up to two folds by this compound (Fig. 8B).

## Discussion

In the current study, we provide several lines of evidence to support our hypothesis that BMS309403 stimulates glucose uptake through selective activation of AMPK signaling pathway. First of all, we demonstrated that BMS309403 significantly increased glucose uptake in myotubes in a time and dose dependent manner, which was paralleled by phosphorylation of AMPKα, p38 and ACC, without involvement of Akt. More direct evidence comes from the fact that inhibition of AMPK by compound C strongly alleviated BMS309403 evoked glucose uptake in C2C12 myotubes. It should also be noted that an additive effect on glucose uptake by BMS309403 and insulin was observed, further supporting our hypothesis that BMS309403 stimulated glucose uptake exclusively through AMPK but not insulin/Akt signaling cascade.

BMS309403 is originally designed as a highly selective inhibitor against FABP4 by occupying the internal lipid binding pocket and competing for the lipid binding in the target proteins [1]. Skeletal muscle and muscle cell lines used in this study are rich in FABP3 but express little FABP4 protein, if at all [13], [14]. In our study, the minimum concentration of BMS309403 required to activate AMPK signaling pathway is 10 µM, which is much higher than the Ki value of 250 nM for FABP3. Thus, it is likely that FABP3 was inhibited under the experimental conditions. However, the effect of BMS309403 on AMPK was not abolished when endogenous FABP3 was knocked down or exogenous hFABP3 was overexpressed, suggesting that BMS309403 evoked AMPK activation and glucose uptake through a mechanism largely independent of FABP3. Therefore it is possible that AMPK dependent glucose uptake by BMS309403 is an off-target effect.

Although BMS309403 is not a direct activator of AMPK, our results indicate that this compound acted as an uncoupler of mitochondrial proton gradient. Interestingly, several anti-diabetic agents, such as troglitazone, rosiglitazone, metformin and berberine, have been shown to activate AMPK via modulation of mitochondrial membrane potential, and thereby exert their pharmacological effects [15], [16]. Chemical uncouplers dissipate proton gradient produced by oxidative phosphorylation, which in turn lead to impaired ATP synthesis, and increased thermogenesis as well. Besides a putative role of brown adipose tissue, in humans, a large part of the thermogenic response is determined by the mitochondrial uncoupling in skeletal muscle [17]. It is suggested that modulation of adaptive thermogenesis by enhancing skeletal muscle mitochondrial uncoupling represents a promising strategy to antagonize obesity and related pathological conditions. Whether BMS309403 increases adaptive themogenesis *in vivo* warrants further investigation, which will provide new insights to establish this class of compound as pharmacological reagents to combat obesity and related diseases.

## Materials and Methods

### Materials

Dulbecco’s modified Eagle’s medium (DMEM) and fetal bovine serum were purchased from Hyclone (Logan, UT, USA). Donor equine serum was purchased from Thermo Scientific (U.S. Origin). Specific antibodies against Acetyl-CoA Carboxylase, Phospho-Acetyl-CoA Carboxylase (Ser79), AMPKα, Phospho-AMPKα (Thr172), Phospho-p38 MAP Kinase (Thr180/Tyr182), Akt, Phospho-Akt (Ser473) were purchased from Cell Signaling Technology (Danvers, MA, USA). Horseradish peroxidase-conjugated secondary antibodies were purchased from Sigma-Aldrich (St. Louis, MO). PVDF membrane was purchased from Millipore (MA, USA). BMS309403 was synthesized in our lab (Institute of Chemical Biology, Guangzhou Institute of Biomedicine and Health, Chinese Academy of Sciences) and dissolved in DMSO in several dilutions. 2-deoxy-D-[^3^H] glucose (37 MBq (1 mCi)) was purchased from GE Healthcare (Shanghai, China). Compound C was purchased from Calbiochem (Darmstadt, Germany). The HIV-based lentiviral vectors and cDNA encoding full-length human FABP3 were purchased from Genecopoeia (Rockville, MD, U.S.). pCMV-3tag vector was purchased from Agilent technologies (Santa Clara, CA, U.S.).

### Cell Culture and Differentiation

Mouse C2C12 skeletal myoblasts (ATCC, USA) were cultured in high glucose DMEM supplemented with 10% (v/v) fetal bovine serum (Hyclone, Logan, UT, USA), 4500 mg/liter glucose, 100 units/ml penicillin and 100 µg/ml streptomycin mixture at 37°C in an incubator with humidified atmosphere of 95% air and 5% CO_2_. Cells were reseeded in 60 mm dishes (for adenine nucleotide analysis), six-well plates (for immunoblotting) or 24-well plates (for glucose uptake). When cells reached confluence, the medium was switched to the differentiation medium (DMEM supplemented with 2% horse serum), which was changed every day. Myotubes were used for experiments 7 days after differentiation.

L6 myoblasts (ATCC, USA) were cultured in high glucose DMEM supplemented with 10% (v/v) fetal bovine serum, 100 units/ml penicillin, and 100 µg/ml streptomycin at CO_2_ incubator (37°C, 5%CO_2_) (Thermo Forma, Marietta, OH, USA). For L6 myoblasts differentiation, the concentration of fetal bovine serum was decreased from 10% to 2%. Myotubes were used for experiments 7 days after differentiation.

### Glucose Uptake in Differentiated C2C12

Differentiated C2C12 cells were starved in serum free-medium for 2 h before incubation with BMS30943. Myotubes were washed twice with glucose free KRPH buffer [140 mM NaCl, 5 mM KCl, 1 mM CaCl_2_, 1.2 mM KH_2_PO_4_, 2.5 mM MgSO_4_, 5 mM NaHCO_3_, 25 mM Hepes, pH 7.4, 0.2% fatty acid free bovine serum albumin], incubated with 0.5 ml of BMS309403 of various concentrations in KRPH buffer for 15 min. The medium was switched to KRPH buffer containing BMS30943, 5 mM D-glucose and 0.5 µCi/well of 2-deoxy-D [^3^H]-glucose for the last 15 min or 5 min. Myotubes were then washed three times with ice-cold PBS and lysed with 0.5 M NaOH and 0.1% SDS. Cell lysates were neutralized with HCl. Radioactivity was measured by liquid scintillation counting (Tri-Carb 2800; Perkin Elmer Co.).

### Western Blotting

Cells were washed twice with ice-cold PBS and lysed with RIPA buffer (50 mM Tris-Cl, pH 7.4, 150 mM NaCl, 5 mM EDTA, 1% Nonidet P-40, 1% sodium deoxycholate, 0.1% SDS, 1% aprotinin, 50 mM NaF, 0.1 mM Na_3_VO_4,_ 1 mM PMSF) for 30 min on ice. Cell lysates were centrifuged at 12,000×g for 15 min at 4°C and supernatants were collected. 30 µg of cellular proteins were resolved by 12% SDS-PAGE gel and transferred to PVDF membrane (Millipore). The membranes were probed overnight with specific antibodies at 4°C, washed three times with TBST followed by incubation with horse radish peroxidase-conjugated secondary antibody for 4 h at 4°C. The membranes were developed by applying ECL Plus developing system (GE Healthcare). Membranes were stripped with 50 mM Tris-HCl, 2%SDS, and 0.1 mM β-mercaptoethanol (pH = 6.8) and reprobed with other antibodies if necessary.

### pCMV-3tag Mediated Overexpression of hFABP3 in C2C12

cDNA encoding full-length human FABP3 was purchased from Genecopoeia (Rockville, MD, U.S.). The hFABP3 cDNA was ligated into pCMV-3tag vector with the 3FLAG tag in the C terminus. The construct was verified by DNA sequencing and used for generation of cell lines. Stable transfectants with pCMV-hFABP3-3tag construct or empty pCMV-3tag vector were selected with 1.5 mg/mL G418 for 10 days. The stable transfectants were clonally picked and switched to differentiation medium (2% horse serum), and cultured for an additional 7 days (myotube) before treatment with BMS309403.

### Lentivirus Mediated Knockdown of FABP3

The HIV-based lentiviral vector was engineered to express EGFP alone or in combination with a specific shRNA sequence of a 19-nucleotide sequence in the coding region of mouse FABP3 (targeting sequence: aggtggctagcatgaccaa) was transfected into C2C12 cells. Stable transfectants were selected with 4 µg/mL puromycin for 14 days. The stable C2C12 tranfectants were clonally picked and differentiated as described before.

### RNA Isolation and Quantitative Real-Time PCR

Total RNA was isolated from cells using Trizol (Invitrogen). First strand complementary DNA was synthesized from 3 µg of total RNA using an oligo(dT)_18_ primer and Superscript III reverse transcriptase (Invitrogen) according to the manufacturer’s instructions. Quantitative PCR was performed on MJ Research Chromo4 Real Time 4-color 96-well PCR system (Bio-Rad) using a SYBR premix *ExTaq* (Takara, Dalian, China.) The PCR primer sets (forward and reverse respectively) used are as follows: FABP3, TCAGCTGGGAATAGAGTTCGAC, TAGTTAGTGTTGTCTCCTGCCC; GAPDH, TGACGTGCCGCCTGGAGAAA, AGTGTAGCCCAAGATGCCCTTCAG. The level of GAPDH was used as reference for data normalization.

### Measurement of AMPK Enzymatic Activity *in vitro*

The method to measure AMPK enzymatic activity *in vitro* was described previously [18]. We chose AMPKα2β1γ1 as the active form and its activity was evaluated by the incorporation of [γ-^33^P] into the SAMS peptide. Radioactivity that had been incorporated in the proteins was determined by liquid scintillation counting in a Wallac MicroBeta TriLus (Wallac, Turku, Finland).

### Mitochondrial Membrane Potential Assay

C2C12 myoblasts were seeded into black 96-well optical-bottom plates at 7,000 cells per well (Greiner Bio-One, Dusseldorf, Germany). On day 5 of differentiation, compounds were added and plates were incubated at 37°C for 1 h. The samples for mitochondrial membrane potential assay were prepared and analyzed as previously described [18]. The ratio of red to green reflected the mitochondrial membrane potential.

### Adenine Nucleotide Extraction and Measurement

C2C12 myotubes cultured in 60 mm dishes were treated with 20 µM BMS309403, washed with PBS and trypsinized. The samples for cellular adenine nucleotides measurements were prepared and analyzed as previously described [18].

### Statistical Analysis

Data were presented as means ± SE. Comparison analysis between groups was performed by using the two-tailed Student’s *t*-test. Differences were considered significant at a *p* value of <0.05.
